# Dietary patterns and non-communicable disease risk in Indian adults: secondary analysis of Indian Migration Study data

**DOI:** 10.1017/S1368980017000416

**Published:** 2017-04-03

**Authors:** Edward JM Joy, Rosemary Green, Sutapa Agrawal, Lukasz Aleksandrowicz, Liza Bowen, Sanjay Kinra, Jennie I Macdiarmid, Andy Haines, Alan D Dangour

**Affiliations:** 1 Department of Population Health, London School of Hygiene & Tropical Medicine, London WC1E 7HT, UK; 2 Leverhulme Centre for Integrative Research on Agriculture and Health (LCIRAH), London, UK; 3 Public Health Foundation of India, Delhi NCR, Institutional Area Gurgaon, India; 4 Department of Non-communicable Disease Epidemiology, London School of Hygiene & Tropical Medicine, London, UK; 5 Public Health Nutrition Research Group, Rowett Institute of Nutrition and Health, University of Aberdeen, Aberdeen, UK; 6 Department of Social & Environmental Health Research, London School of Hygiene & Tropical Medicine, London, UK

**Keywords:** Dietary patterns, Finite mixture modelling, Indian Migration Study, Micronutrient malnutrition, Non-communicable disease risk factors

## Abstract

**Objective:**

Undernutrition and non-communicable disease (NCD) are important public health issues in India, yet their relationship with dietary patterns is poorly understood. The current study identified distinct dietary patterns and their association with micronutrient undernutrition (Ca, Fe, Zn) and NCD risk factors (underweight, obesity, waist:hip ratio, hypertension, total:HDL cholesterol, diabetes).

**Design:**

Data were from the cross-sectional Indian Migration Study, including semi-quantitative FFQ. Distinct dietary patterns were identified using finite mixture modelling; associations with NCD risk factors were assessed using mixed-effects logistic regression models.

**Setting:**

India.

**Subjects:**

Migrant factory workers, their rural-dwelling siblings and urban non-migrants. Participants (7067 adults) resided mainly in Karnataka, Andhra Pradesh, Maharashtra and Uttar Pradesh.

**Results:**

Five distinct, regionally distributed, dietary patterns were identified, with rice-based patterns in the south and wheat-based patterns in the north-west. A rice-based pattern characterised by low energy consumption and dietary diversity (‘Rice & low diversity’) was consumed predominantly by adults with little formal education in rural settings, while a rice-based pattern with high fruit consumption (‘Rice & fruit’) was consumed by more educated adults in urban settings. Dietary patterns met WHO macronutrient recommendations, but some had low micronutrient contents. Dietary pattern membership was associated with several NCD risk factors.

**Conclusions:**

Five distinct dietary patterns were identified, supporting sub-national assessments of the implications of dietary patterns for various health, food system or environment outcomes.

India faces a double burden of malnutrition: dietary deficiency of energy and nutrients are widespread particularly among poorer, rural populations^(^ ^1^ ^)^, while non-communicable diseases (NCD) related to excessive energy, fat, salt and sugar consumption and reduced levels of physical activity are increasingly prevalent, especially among urban populations^(^ ^2^ ^–^ ^4^ ^)^. The Global Burden of Disease study estimated that 1081 disability-adjusted life-years per 100000 population were lost in India in 2013 due to deficiencies of Fe, Zn and vitamin A, while 2489 disability-adjusted life-years per 100000 population were lost due to high serum total cholesterol or BMI^(^ ^5^ ^)^. An estimated 20 % of men and 21 % of women aged ≥20 years were obese in 2013 using South Asian-specific obesity cut-offs^(^ ^6^ ^,^ ^7^ ^)^.

Identifying robust and plausible associations between diets and health outcomes could help to guide agriculture, nutrition and public health policy development. Diets in India are however extremely diverse due to various geographic, cultural, social and economic factors, making it more appropriate to define and study sub-national dietary patterns rather than a national average diet. Previous attempts to characterise dietary patterns in India have several shortcomings in terms of data availability or analysis methods^(^ ^8^ ^)^ and have typically focused either on undernutrition or NCD risks but not both.

In the present study we examined dietary patterns and associated health outcomes among Indian adults based on a large multi-state survey of urban migrants and their rural-dwelling siblings. The primary aim of the study was to identify distinct dietary patterns among the study population using finite mixture modelling. The secondary aim was to examine the association of the identified dietary patterns with macro- and micronutrient intakes and five key NCD risk factors: BMI, waist:hip ratio (WHR), systolic or diastolic blood pressure, serum cholesterol (total:HDL) and fasting blood glucose.

## Methods

### Participants and setting

The Indian Migration Study (IMS) was a cross-sectional, sibling-pair comparison study conducted around four factories situated in northern (Lucknow), central (Nagpur, Hyderabad) and southern (Bangalore) India during 2005–2007^(^ ^9^ ^)^. Factory workers and their co-resident spouses were surveyed to establish their migration status. Rural-to-urban migrants, their non-migrant sibling still residing in the place of origin and a 25 % random sample of urban non-migrants were recruited to the study. Siblings were preferably of the same sex and closest in age; cousins or close friends were recruited if siblings were unavailable. A total of 7067 individuals were included in the final sample^(^ ^10^ ^,^ ^11^ ^)^.

### Dietary intake and food composition data

Dietary intake was assessed using an interviewer-administered semi-quantitative FFQ^(^ ^12^ ^)^. Participants reported the portion size and frequency of consumption of up to 184 meals or food items over the past year^(^ ^13^ ^,^ ^14^ ^)^. For fruit and vegetable items, participants were asked whether their consumption was seasonal and how much they consumed when the item was in season. To quantify average consumption over the year, this value was multiplied by the proportion of the year for which the item was in season, determined through a survey of local market vendors. The FFQ included commonly consumed dishes for which weighed recipes were generated for rural and urban areas of the four study sites. These recipe sheets were used to calculate individual intake of 201 distinct food items; these food items were aggregated into thirty-six food groups based on similarity in nutritional content (see online supplementary material, Supplemental Table 1).

**Table 1:** Socio-economic characteristics of respondents in the Indian Migration Study (2005–2007) by dietary pattern

	‘Rice & low diversity’		‘Rice & fruit’		‘Wheat & pulses’		‘Wheat, rice & oils’		‘Rice & meat’		Total	
	*n*	%	*n*	%	*n*	%	*n*	%	*n*	%	*n*	%
Total	1339	19·8	1505	22·2	1953	28·8	1462	21·6	516	7·6	6775	100·0
Sex												
Males	784	58·5	724	48·1	1172	60·0	888	60·7	312	60·5	3880	57·3
Females	555	41·5	781	52·9	781	40·0	574	39·3	204	39·5	2895	42·7
Age (years)												
<30	83	6·2	81	5·4	138	7·1	696	47·6	51	9·9	1049	15·5
30–40	304	22·7	347	23·1	423	21·7	601	41·1	120	23·3	1795	26·5
40–50	536	40·0	663	44·1	879	45·0	132	9·0	186	36·0	2396	35·4
>50	416	31·1	414	27·5	513	26·3	33	2·3	159	30·8	1535	22·7
Region												
North	6	0·5	6	0·4	1837	94·1	178	12·2	9	1·7	2036	30·0
East	11	0·8	5	0·3	54	2·8	52	3·6	3	0·6	125	1·8
South	1302	97·2	1477	98·2	11	0·5	31	2·1	471	91·3	3292	48·6
West	20	1·5	17	1·1	51	2·6	1201	82·1	33	6·4	1322	19·5
Location												
Urban	573	42·8	1163	77·3	1346	68·9	910	62·2	277	53·7	4269	63·0
Rural	766	57·2	342	22·7	607	31·1	552	37·8	239	46·3	2506	37·0
Marital status												
Married	1206	90·1	1387	92·2	1802	92·3	1092	74·7	457	88·6	5944	87·7
Unmarried	133	9·9	118	7·8	151	7·7	370	25·3	59	11·4	831	12·3
Education												
None	325	24·3	92	6·1	173	8·9	53	3·6	123	23·8	766	11·3
Primary	299	22·3	229	15·2	198	10·1	84	5·8	102	19·8	912	13·5
Secondary	557	41·6	729	48·5	749	38·3	983	67·2	230	44·6	3248	47·9
Tertiary	158	11·8	455	30·2	833	42·7	342	23·4	61	11·8	1849	27·3
Occupation												
None	447	33·4	614	40·8	732	37·5	613	41·9	165	32·0	2571	38·0
Unskilled manual	306	22·8	114	7·6	353	18·1	274	18·7	108	20·9	1155	17·1
Skilled manual	296	22·1	330	21·9	201	10·3	482	33·0	127	24·6	1436	21·2
Non-manual	229	17·1	203	13·5	557	28·5	46	3·2	93	18·0	1128	16·7
Professional	61	4·6	244	16·2	110	5·6	47	3·2	23	4·5	485	7·2
Religion												
Hindu	1216	90·8	1383	91·9	1799	92·1	1342	91·8	434	84·1	6174	91·1
Other	123	9·2	122	8·1	154	7·9	120	8·2	82	15·9	601	8·9
Own agricultural land												
Yes	692	51·7	533	35·4	691	35·4	545	37·3	233	45·2	2692	39·8
No	647	48·3	972	64·6	1262	64·6	917	62·7	283	54·8	4081	60·2

The FFQ was repeated 1–2 months and 12 months after initial collection in a sub-sample of participants to check reliability, while the FFQ was validated by administering three 24 h recalls in a sub-sample of 530 participants. Reported energy consumption was on average 1711 kJ/d greater in the FFQ than the 24 h recall but the FFQ data were deemed valid for comparison between groups^(^ ^12^ ^)^. Data on sociodemographic factors, anthropometry and biochemical risk factors were also collected^(^ ^10^ ^,^ ^15^ ^)^.

The nutrient composition of the 201 distinct food items was derived from Indian food composition tables^(^ ^16^ ^)^ and US composition tables^(^ ^17^ ^)^ where data from India were unavailable. Average nutrient composition of the thirty-six food groups was calculated as the average composition of constituent items weighted by the mean consumption of items across the study population. The SFA and PUFA composition of meals was specific to the type of cooking oil used by each household.

### Defining dietary patterns

The FFQ method estimated mean energy intake as 12129 (sd 4192) kJ/capita per d. The FFQ method is liable to misreporting^(^ ^18^ ^)^ and we removed individuals with extreme daily energy intake defined as mean±2sd ^(^ ^19^ ^)^. Individuals consuming >20510 kJ/d (*n* 272) or <3749 kJ/d (*n* 20) were excluded, leaving 6775 observations in the data set.

For each individual and food group, consumption was categorised into four levels, i.e. zero consumption and tertiles of energy from that food group as a proportion of total dietary energy consumption. The thirty-six categorical variables representing proportional consumption of food groups were entered into a mixture model using latent class analysis^(^ ^20^ ^)^, to identify distinct patterns of food consumption. Solutions containing one to ten distinct dietary patterns were specified; we used a combination of diagnostic criteria (Bayesian information criterion, minimum proportion per class and entropy of model)^(^ ^21^ ^)^ to select the solution that fitted the data best. Individuals were assigned to the dietary patterns based on probability.

### Dietary patterns, nutrition and health

Dietary supply of nutrients was calculated for each individual as the product of daily food group consumption and mean food group composition. We described the nutritional profile of each dietary pattern and compared these with WHO guidelines for macronutrients^(^ ^22^ ^)^ and micronutrients^(^ ^23^ ^)^.

We constructed mixed-effects multiple regression models to investigate whether dietary patterns were associated with five key NCD risk factors: BMI, WHR, systolic blood pressure or diastolic blood pressure, serum total:HDL cholesterol and fasting blood glucose. Analytical procedures were reported previously^(^ ^10^ ^,^ ^15^ ^)^. We analysed binary outcomes as appropriate for South Asian populations. Thus: BMI<18·5 kg/m^2^ and BMI≥25·0 kg/m^2^ were classed as ‘underweight’ and ‘obese’, respectively^(^ ^6^ ^,^ ^24^ ^)^; WHR>0·9 for men and WHR>0·8 for women were classed as detrimental to health^(^ ^25^ ^)^; systolic blood pressure ≥140 mmHg or diastolic blood pressure ≥90 mmHg was classed as hypertensive^(^ ^26^ ^)^; serum total:HDL cholesterol ≥4·5 was classed as detrimental to health^(^ ^27^ ^–^ ^29^ ^)^; and fasting blood glucose ≥7·0 mmol/l indicated diabetes^(^ ^30^ ^)^. Participants may have taken medication or adapted dietary or lifestyle choices in response to a known condition, so those self-reporting diabetes and hypertension were included as cases.

Our models took account of the sib-pair clustered study design by including sib-pair as a family-specific random effect. A causal path diagram helped to visualise the relationships between dietary patterns and risk factors and assisted in construction of the models. Dietary pattern membership was treated as a predictor of each risk factor in turn, while adjusting for the potential confounding factors of age, sex, education level and rural/urban residency. The following factors were also considered as potential confounders for specific outcomes: energy consumption by quartile for underweight, obesity and WHR; obesity for hypertension, total:HDL cholesterol and diabetes; and current smoking status for hypertension. An age-squared term was included for underweight and obesity. Models were tested for multicollinearity using a correlation matrix of bivariate relationships between explanatory variables and the variance inflation factor for each regression model. Statistical analyses were conducted in MPlus version 7.3 (Muthén & Muthén, Los Angeles, CA, USA) for the mixture models and in Stata version 14 and R version 3.2.2 for descriptive statistics and regression models.

## Results

### Participant and dietary pattern characteristics

Over half of study participants were male (57 %), while the majority were married (88 %) and identifying as Hindu (91 %; Table 1). If states are allocated to ‘major regions’^(^ ^31^ ^)^, almost half of the respondents (49 %) were from the South, 30 % from the North and 20 % from the West. Only 2 % of respondents were from the East. The mean age of respondents was 41 (sd 10) years (range 17–76 years).

**Fig. 1:**
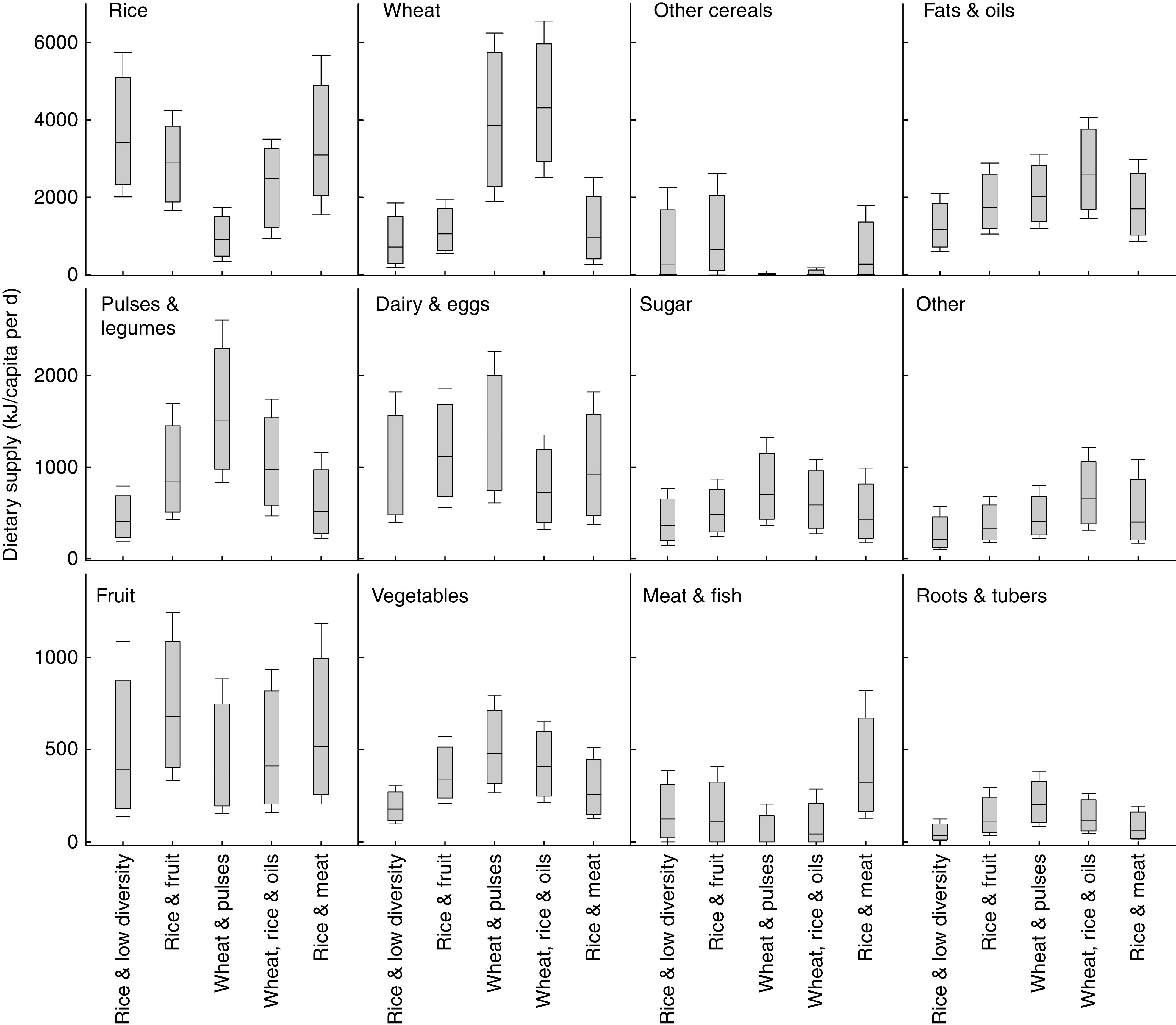
Box-and-whisker plots showing food group consumption of respondents in the Indian Migration Study (2005–2007) by dietary pattern. The bottom and top edge of the box represent the first and third quartiles (interquartile range); the line within the box represents the median; and the ends of the bottom and top whiskers represent the 10th and 90th percentiles, respectively

The results of the mixture modelling showed that a five-pattern solution best described the data according to model fit. The probability of correct pattern assignment was >90 % for all individuals. The five-pattern solution also appeared to describe the data well with clear distinctions in consumption of different food groups and this was apparent when visualising consumption of aggregated food groups (Fig. 1 and online supplementary material, Supplemental Table 2). We named the patterns by the main staple grain(s) and one other identifying feature, as follows: ‘Rice & low diversity’; ‘Rice & fruit’; ‘Wheat & pulses’; ‘Wheat, rice & oils’; and ‘Rice & meat’.

**Table 2:** Summary of mean nutrient consumption among adults in the Indian Migration Study (2005–2007) compared with WHO adult guidelines by dietary pattern. Guideline values for dietary micronutrient supplies are Estimated Average Requirements^(^ ^23^ ^)^

	Energy									Males (18–60 years)			Females (18–60 years)		
	(kJ/capita per d)	Fat (%E)	SFA (%E)	PUFA (%E)	Protein (%E)	CHO (%E)	Cholesterol (g/d)	Na (g/d)	F&V (g/d)	Ca (mg/d)	Fe (mg/d)	Zn (mg/d)	Ca (mg/d)	Fe (mg/d)	Zn (mg/d)
WHO guidelines	–	15–30	<10	6–10	10–15	55–75	<0·3	<2	>400	625	10·5	11·7	625	18·4	8·2
Dietary pattern															
‘Rice & low diversity’	9912	23	7·2	6·6	10	67	0·121	3·7	271	909	17·9	10·0	736	14·5	8·0
‘Rice & fruit’	11556	28	8·5	8·0	11	62	0·134	5·3	472	1151	22·8	11·9	976	19·2	10·0
‘Wheat & pulses’	12665	27	8·3	8·2	12	61	0·112	4·3	518	1179	24·3	10·7	1038	22·3	9·7
‘Wheat, rice & oils’	13991	27	6·4	9·3	11	62	0·096	4·7	434	959	28·9	11·1	762	23·7	9·1
‘Rice & meat’	11393	27	7·6	8·0	11	62	0·206	4·8	369	1005	22·4	11·8	878	19·7	10·2
All	12062	26	7·7	8·1	11	62	0·123	4·5	430	1055	23·6	11·0	897	20·0	9·4
Proportion (%) missing target*															
‘Rice & low diversity’	–	11	10	41	36	7	4	90	84	24	9	73	42	80	58
‘Rice & fruit’	–	30	20	10	24	0	4	100	39	6	2	55	13	51	31
‘Wheat & pulses’	–	27	18	7	3	0	3	97	31	6	2	65	10	27	29
‘Wheat, rice & oils’	–	25	2	3	23	0	3	96	46	13	1	57	39	25	41
‘Rice & meat’	–	28	13	16	13	3	15	97	63	18	3	52	30	47	30
All	–	24	13	14	19	2	4	96	49	12	3	62	24	45	37

%E, percentage of total energy; CHO, carbohydrate; F&V, fruit and vegetables.

* For the proportion of individuals missing the target intake, targets were set as the maximum of range for fat, SFA and CHO; minimum of range for PUFA and protein; and on an individual level on the basis of age and sex for mineral micronutrients.

Region was the strongest predictor of pattern membership, with participants from the South region tending to consume rice-based patterns while participants from the North and West regions tended to consume wheat-based patterns (Table 1). Religion was also important, with a greater proportion of non-Hindus in the ‘Rice & meat’ pattern. Among consumers of the ‘Rice & low diversity’ pattern, 57 % lived in rural areas compared with 37 % of the sample population. The ‘Rice & low diversity’ pattern is probably the closest representation of a ‘pre-nutrition transition’ diet and we therefore used it as the reference pattern for the epidemiological analysis. Consumers of the mixed ‘Wheat, rice & oils’ pattern had a younger age distribution, i.e. mean of 30 years compared with 44 years for other patterns.

### Characteristics of dietary patterns

The ‘Rice & low diversity’ pattern had the lowest energy supply, i.e. 9912 (sd 3180) kJ/capita per d compared with the overall mean of 12062 (sd 3732) kJ/capita per d. Consumption of other food groups including fruits and vegetables was low (Table 2, Fig. 1). Mean consumption of fruit in the ‘Rice & fruit’ pattern was 761 (sd 423) kJ/capita per d (or 186 (sd 106) g/capita per d) compared with the overall mean of 561 (sd 402) kJ/capita per d (or 149 (sd 103) g/capita per d). The mean consumption of pulses and legumes in the ‘Wheat & pulses’ pattern was 1619 (sd 707) kJ/capita per d compared with an overall mean of 1050 (sd 686) kJ/capita per d. Vegetable consumption was greater and fruit consumption lower than in the rice-based patterns. The mixed ‘Wheat, rice & oils’ pattern had the greatest mean total energy consumption, i.e. 13991 (sd 3632) kJ/capita per d. A distinct feature of this pattern was the high consumption of both rice and wheat, with mean consumption of 2326 (sd 1079) kJ/capita per d and 4427 (sd 1607) kJ/capita per d, respectively. The mean consumption of fats and oils in the ‘Wheat, rice & oils’ pattern was 2703 (sd 992) kJ/capita per d compared with the overall mean of 2000 (sd 933) kJ/capita per d. Finally, meat and fish consumption was greatest in the ‘Rice & meat’ pattern, i.e. mean of 427 (sd 368) kJ/capita per d compared with an overall mean of 146 (sd 226) kJ/capita per d.

### Dietary patterns, nutrition and health

The mean balance of macronutrients was within the WHO guidelines for all five dietary patterns (Table 2); however, the proportion of individuals outside the recommended ranges varied between patterns, as did the proportion of individuals with micronutrient intake below the Estimated Average Requirement. For example, in the ‘Rice & fruit’ pattern 30 % of participants derived >30 % of energy from fat and 13 % of females had inadequate Ca intake; whereas in the ‘Rice & low diversity’ pattern 11 % of participants derived >30 % of energy from fat and 42 % of females had inadequate Ca intake. In addition, 49 % of individuals did not meet the recommended 400 g/d consumption of fruits and vegetables, rising to 84 % among the ‘Rice & low diversity’ pattern. Mean levels of dietary Na and cholesterol also varied between dietary patterns (Table 2). All dietary patterns exceeded the recommended Na intake level and the intake of saturated fat was greater than recommended for 20 % of consumers of the ‘Rice & fruit’ pattern.

Mean (unadjusted) levels of NCD risk factors differed between the dietary patterns (Table 3) and suggested that despite its high total energy content, consumers of the mixed ‘Wheat, rice & oils’ pattern had the most favourable health profile. However, consumers of this pattern were typically younger and associations were explored further in mixed-effects logistic regression models (Table 4). In fully adjusted models, compared with the reference ‘Rice & low diversity’ dietary pattern, consumers of the ‘Wheat, rice & oils’ pattern had the greatest odds of being underweight (OR=3·48; 95 % CI 2·46, 4·92) and the lowest odds of being obese (OR=0·43; 95 % CI 0·33, 0·56). Consumers of the ‘Wheat & pulses’ pattern had raised odds of a high WHR (OR=1·23; 95 % CI 1·01, 1·51). Consumers of the ‘Rice & fruit’ pattern had the greatest odds of obesity (OR=1·19; 95 % CI 0·97, 1·46) and reduced odds of hypertension (OR=0·72; 95 % CI 0·58, 0·90). In fully adjusted models, dietary pattern was not significantly associated with an unhealthy total:HDL cholesterol or diabetes.

**Table 3:** Mean levels of non-communicable disease risk factors among adults in the Indian Migration Study (2005–2007) by dietary pattern

	BMI (kg/m^2^)		WHR		Systolic blood pressure (mmHg)		Serum total:HDL cholesterol		Fasting blood glucose (mmol/l)	
Dietary pattern	Mean	sd	Mean	sd	Mean	sd	Mean	sd	Mean	sd
‘Rice & low diversity’	24·0	4·4	0·869	0·078	126	19	4·4	1·1	5·2	1·1
‘Rice & fruit’	25·5	4·2	0·867	0·089	124	18	4·5	1·2	5·3	1·4
‘Wheat & pulses’	24·3	4·4	0·886	0·088	123	17	4·4	1·5	5·7	1·7
‘Wheat, rice & oils’	21·2	3·6	0·854	0·070	114	12	4·1	1·3	4·8	1·1
‘Rice & meat’	24·4	4·8	0·881	0·083	128	20	4·5	1·3	5·2	1·1
Overall	23·8	4·5	0·871	0·083	122	17	4·4	1·3	5·3	1·4

WHR, waist:hip ratio.

**Table 4:** Dietary pattern as a predictor of non-communicable disease risk factors among adults in the Indian Migration Study (2005–2007), determined using mixed effects logistic regression models

	Underweight*			Obesity†			WHR‡			Hypertension§			Total:HDL cholesterol║			Diabetes¶		
	(*N* 6770)			(*N* 6770)			(*N* 6763)			(*N* 6769)			(*N* 6680)			(*N* 6751)		
Dietary pattern	OR	95 % CI	*n*	OR	95 % CI	*n*	OR	95 % CI	*n*	OR	95 % CI	*n*	OR	95 % CI	*n*	OR	95 % CI	*n*
‘Rice & low diversity’	1	–	138	1	–	513	1	–	753	1	–	414	1	–	539	1	–	127
‘Rice & fruit’	0·70	0·48, 1·03	59	1·19	0·97, 1·46	801	1·09	0·89, 1·34	944	0·72**	0·58, 0·90	426	1·13	0·92, 1·39	666	1·08	0·80, 1·46	188
‘Wheat & pulses’	2·13**	1·56, 2·92	192	0·72**	0·59, 0·89	810	1·23**	1·01, 1·51	1255	0·82	0·66, 1·01	553	0·89	0·73, 1·10	767	1·21	0·90, 1·62	245
‘Wheat, rice & oils’	3·48**	2·46, 4·92	364	0·43**	0·33, 0·56	225	1·03	0·82, 1·30	656	0·55**	0·41, 0·74	110	0·92	0·72, 1·17	467	1·09	0·72, 1·66	57
‘Rice & meat’	0·86	0·55, 1·34	43	1·13	0·86, 1·49	214	1·25	0·96, 1·63	320	1·29	0·98, 1·70	182	1·16	0·89, 1·52	224	1·34	0·92, 1·98	65

WHR, waist:hip ratio; *N*, number of population; *n*, number of cases. The reference diet is the ‘Rice & low diversity’ pattern. All models controlled for age, sex, rural/urban residency and education level of participants and included sib-pair as a family-specific random effect. Additional potential confounders included in the models are listed in the footnotes below.

* BMI<18·5 kg/m^2^. Also controlled for: age-squared and energy consumption quartile.

† BMI≥25·0 kg/m^2^. Also controlled for: age-squared and energy consumption quartile.

‡ Unhealthy WHR defined as >0·9 for men and >0·8 for women. Also controlled for: energy consumption quartile.

§ Systolic blood pressure ≥140 mmHg or diastolic blood pressure ≥90 mmHg. Also controlled for: obesity and smoking.

║ Unhealthy total:HDL cholesterol defined as ≥4·5. Also controlled for: obesity.

¶ Fasting blood glucose ≥7·0 mmol/l. Also controlled for: obesity.

** ≥95 % confidence that the OR is not equal to 1.

## Discussion

### Principal findings

The present study aimed to define distinct, typical dietary patterns among adults participating in the IMS and investigate associations with NCD risk factors and micronutrient intakes. Using finite mixture models, we identified five distinct dietary patterns that represented different sub-populations and had diverse nutrient content. Our analysis identified three rice-based patterns, one wheat-based pattern and one pattern that contained both rice and wheat as staple foods. The patterns reflect well-established culturally and geographically relevant dietary preferences in India (i.e. rice-based diets in the South; wheat-based dies in the North and West) but also credible socio-economic differences in dietary habits such as a low-diversity rice-based diet consumed by poorer, rural adults and a mixed rice and wheat diet with oils consumed by younger, urban adults. In some cases, the dietary patterns identified were significantly associated with several NCD risk factors after controlling for sociodemographic variables, demonstrating the utility of dietary pattern analysis in identifying diet-related health risks.

Potential mechanisms through which dietary patterns influenced NCD risk factors can be proposed, although further studies are required to validate these. For example, consumers of the ‘Wheat & pulses’ pattern may have had lower odds of hypertension due to greater consumption of pulses^(^ ^32^ ^)^, but greater odds of diabetes due to greater consumption of sugar^(^ ^33^ ^)^. Consumers of the ‘Wheat & pulses’ pattern had raised odds of a high WHR but lower odds of obesity. This may be an important finding considering the particular risk factors associated with diabetes among Asian Indians^(^ ^34^ ^)^, and consumers of this pattern had greater odds of diabetes, although this was not significant at the 95 % level. Similarly, consumers of the ‘Rice & meat’ pattern had greater odds of a high WHR, which was significant at the 95 % level if energy intake was not controlled for (see online supplementary material, Supplemental Table 3), and greater odds of diabetes, although this was not significant at the 95 % level. Greater dietary diversity including fruit and vegetable consumption may have reduced risks of hypertension among consumers of the ‘Rice & fruit’, ‘Wheat & pulses’ and ‘Wheat, rice & oils’ patterns^(^ ^35^ ^)^.

Consumers of the ‘Wheat, rice & oils’ pattern had the greatest odds of being underweight and the lowest odds of obesity, despite having the greatest energy consumption (although this was controlled for in the analyses). This apparently anomalous result may be due to the distinctively younger age profile of consumers of this pattern, perhaps indicating that the models were under-adjusted for age or physical activity levels. There was also evidence of an interaction effect between age, BMI and dietary pattern as indicated by the different trajectories of BMI *v.* age by dietary pattern (see online supplementary material, Supplemental Fig. 1). Energy intake was controlled for to distinguish the effects of dietary patterns on the outcomes underweight, obesity and WHR, independent of energy intakes. However, energy intakes are connected to body size and physical activity level, so this may be an over-adjustment. As a sensitivity analysis, we re-ran the regression analyses without controlling for energy intakes. For all dietary patterns in comparison to the reference, the odds ratios were similar but slightly smaller for underweight, and similar but slightly larger for obesity and WHR (Supplemental Table 3). For consumers of the ‘Rice & meat’ pattern, the lower bound of the 95 % CI of WHR was now >1.

Longitudinal data would help establish whether the association between age and BMI was a life course, period or cohort effect.

### Study limitations

There are two primary limitations in our work. First, the IMS was designed to study the influence of rural-to-urban migration on the health of adults and the population is not nationally representative, e.g. 37 % of the IMS population living in rural areas compared with 70 % in the whole of India in 2006^(^ ^36^ ^)^. Furthermore, participants were recruited from four factory settings which led to geographic clustering, e.g. <2 % of participants were drawn from the East region. While this limits the ability to interpret our findings at a national level, we have met our main aims to identify typical dietary patterns and their association with health within this large data set. In addition, the IMS data were collected in 2005–2007 and dietary patterns may have changed subsequently.

The second main limitation relates to the reliability of FFQ data. Estimated energy intakes were 19 % greater in the IMS FFQ compared with 24 h recalls^(^ ^12^ ^)^ and misreporting may have affected some food items more than others. Notably, the mean of reported energy consumption in the ‘Wheat, rice & oils’ pattern was 13991 kJ/capita per d, which is greater than the upper 2·5th percentile of energy consumption reported via dietary recall in the UK^(^ ^37^ ^)^. Thus, the proportion of individuals with inadequate intakes of micronutrients may be underestimated and intakes of fat and other nutrients overestimated. However, 24 h recalls are also susceptible to misreporting, particularly under-reporting^(^ ^38^ ^)^. Furthermore, our methods to identify dietary patterns relied on relative consumption patterns rather than on absolute consumption quantities and this may have reduced the errors associated with misreporting in dietary surveys.

Other limitations were as follows. First, food composition data were derived from a study published in 1971. The accuracy and relevance of these data could be improved through spatially refined analysis of modern crop varieties using the latest analytical procedures^(^ ^39^ ^,^ ^40^ ^)^. Second, NCD risk factors were measured at one point in time only; repeat measures would be preferable. Third, participants may have adapted their dietary choices for a known condition, raising the possibility of reverse causality. Fourth, there was potential residual confounding in the regression model, e.g. due to the binary categorisation of some confounding variables. Fifth, the number of individuals with diabetes was relatively small and results of the regression models should be interpreted with caution. Sixth, physical activity was not controlled for, partly because this was not reliably captured in the IMS and partly because it is likely to be strongly correlated with age, rural/urban residency, education level (and therefore occupation) and energy consumption; however, this might have led to under-adjustment for physical activity. Finally, we did not control for alcohol consumption which is a known risk factor for obesity, hypertension and diabetes. The quantity and frequency of alcohol consumption are important but alcohol consumption was recorded in the IMS only as ‘never’, ‘current (consumed within last 6 months)’ and ‘previous (stopped >6 months ago)’. In addition, alcohol consumption was associated with ‘upstream’ variables included in the model such as age, sex and education level as well as dietary pattern membership, and was therefore excluded from the analysis.

### Comparison with other studies

Most dietary pattern analyses for India have used principal component analysis, finding two to six distinct dietary patterns in various large data sets^(^ ^8^ ^)^. The majority of patterns were defined by vegetarian food groups, e.g. Satija *et al*.^(^ ^41^ ^)^ identified three dietary patterns in the IMS data with one ‘animal food’ pattern. The importance of fish in defining dietary patterns in eastern India has been reported in previous studies^(^ ^8^ ^)^, yet the East region of India was under-represented in the IMS.

Mixture modelling such as LCA provides a number of benefits over principal component analysis, namely the ability to calculate the mean consumption of each food group in each pattern and to allocate each individual to a single dietary pattern based on probability^(^ ^42^ ^)^. Thus, we were able to quantify the nutritional content of typical dietary patterns and compare with WHO dietary guidelines. A further strength of the current study is the quantification of aspects relating to both undernutrition and diet-related NCD. This approach was taken specifically to reflect the existence of the double burden of malnutrition in the Indian context. Similar to previous studies, we found evidence of an association between dietary pattern and body size^(^ ^41^ ^,^ ^43^ ^–^ ^45^ ^)^, including prevalence of underweight and obesity. Unlike previous studies^(^ ^46^ ^)^, we found no evidence that vegetarian diets incur a lower risk of diabetes.

### Policy relevance and research needs

Although the present study is cross-sectional, comparing patterns may provide an insight into the dietary changes. For example, the ‘Rice & low diversity’ and ‘Rice & fruit’ patterns represent predominantly rural and urban populations, respectively, from the South region. Moving from the rural to the urban pattern there is an increase in overall energy and salt consumption, a decline in the proportion of dietary energy derived from rice and an increase in the proportion of dietary energy from fats and oils, fruit, pulses and legumes. Consumers of the urban pattern had greater odds of obesity, but lower odds of underweight, hypertension or dietary Ca, Fe and Zn deficiencies (Tables 2 and 4). Thus, there are likely to be beneficial and detrimental impacts on health due to dietary changes in India. An improved understanding of the links between dietary patterns and health may help to guide policies such as public education about diets and nutrition, strategies to improve access to healthy foods and investments in health care in preparation for changing disease profiles in the population. The results of the current study are indicative of the links between typical dietary patterns and health in India, but complementary studies are required including with more recent and nationally representative dietary data.

Previous studies have reported that India is undergoing a nutrition transition^(^ ^4^ ^)^. In many other countries where nutrition transitions have occurred or are underway, consumption of meat has increased greatly, e.g. from 68 to 169 g/capita per d between 1990 and 2013 in China^(^ ^47^ ^)^. Mean meat and fish consumption was low in the present study, i.e. 27 (sd 42) g/capita per d, but was greater in the ‘Rice & meat’ pattern, i.e. 78 (sd 69) g/capita per d. Compared with the overall population, consumers of the ‘Rice & meat’ pattern had a similar profile of occupations and were more likely to be living in rural areas. Thus, whereas meat consumption rose steeply with urbanisation and greater incomes in China, the association in India is likely to be mediated by cultural factors, in particular the preference for vegetarian or lacto-ovo-vegetarian diets among many Hindus.

Determining associations between dietary patterns and risks of disease may help in the development or targeting of public nutrition and health strategies. Thus, future work could apply the methodology to other large sample populations in India including those with more recent dietary data.

## Supplementary material

For supplementary material accompanying this paper visit https://doi.org/10.1017/S1368980017000416. click here to view supplementary material
